# Trends and impacts of SARS-CoV-2 genome sharing: a comparative analysis of China and the global community, 2020–2023

**DOI:** 10.3389/fpubh.2024.1491623

**Published:** 2024-11-20

**Authors:** Yenan Feng, Songqi Chen, Anqi Wang, Zhongfu Zhao, Cao Chen

**Affiliations:** National Key Laboratory of Intelligent Tracking and Forecasting for Infectious Diseases, NHC Key Laboratory of Medical Virology and Viral Diseases, National Institute for Viral Disease Control and Prevention, Chinese Center for Disease Control and Prevention, Beijing, China

**Keywords:** SARS-CoV-2, genomic sequence, data sharing, GISAID, China

## Abstract

**Objective:**

The global sharing of pathogen genome sequences has been significantly expedited by the COVID-19 pandemic. This study aims to elucidate the global landscape of SARS-CoV-2 genome sharing between 2020 and 2023 with a focus on quantity, timeliness, and quality. Specifically, the characteristics of China are examined.

**Methods:**

SARS-CoV-2 genomes along with associated metadata were sourced from GISAID database. The genomes were analyzed to evaluate the quantity, timeliness, and quality across different countries/regions. The metadata characteristics of shared genomes in China in 2023 were examined and compared with the actual demographic data of China in 2023.

**Results:**

From 2020 to 2023, European countries consistently maintained high levels of genomic data sharing in terms of quantity, timeliness, and quality. In 2023, China made remarkable improvements in sequence sharing, ranking among the top 3.89% globally for quantity, 22.78% for timeliness, and 17.78% for quality. The genome sharing in China in 2023 covered all provinces with Shanghai Municipality contributing the most genomes. Human samples accounted for 99.73% of the shared genomes and exhibited three distinct peaks in collection dates. Males constituted 52.06%, while females constituted 47.94%. Notably, there was an increase in individuals aged 65 and above within the GISAID database compared to China’s overall population in 2023.

**Conclusion:**

The global sharing of SARS-CoV-2 genomes in 2020–2023 exhibited disparities in terms of quantity, timeliness, and quality. However, China has made significant advancements since 2023 by achieving comprehensive coverage across provinces, timely dissemination of data, and widespread population monitoring. Strengthening data sharing capabilities in countries like China during the SARS-CoV-2 pandemic will play a crucial role in containing and responding to future pandemics caused by emerging pathogens.

## 1 Introduction

The timely sharing of genomic sequences and associated metadata has played a crucial role in promoting global data awareness, enhancing our understanding of pathogenic evolution characteristics, and facilitating the development of detection reagents, vaccines, and drugs (1–4), which was particularly evident during the COVID-19 pandemic (5, 6). Since January 10, 2020, when China released the initial genome sequence of SARS-CoV-2 into the Global Initiative on Sharing All Influenza Data (GISAID) database, more than 16 million SARS-CoV-2 genomes have been stored in GISAID to date (7). In contrast, GenBank, serves as another prominent repository, hosted a collection of over 8 million SARS-CoV-2 genomes (8). It encompasses all other International Nucleotide Sequence Database Collaboration (INSDC) databases (9), including the European Nucleotide Archive (ENA) (10) and the DNA Data Bank of Japan (DDBJ) (11). Other repositories, such as the China National Center for Bioinformation GenBase (12), have stored more than 40,000 SARS-CoV-2 genomes. The China National GeneBank DataBase (CNGBdb) (13), along with the Novel Coronavirus National Science and Technology Resource Service System at the National Microbiology Data Center, has, respectively, collected a small number of 87 and 305 SARS-CoV-2 genomes (9). Several analysis platforms such as Outbreak. Info (14), Cov-Spectrum.org (15), and CoVariants.org (16) were opportunistically developed utilizing these publicly available datasets. Additionally, there have been numerous intriguing investigations conducted based on the sharing of SARS-CoV-2 data to further explore the virus’s structure, pathogenic mechanisms, mutation biases, and more (17–23). The World Health Organization (WHO) Guiding Principles for Pathogen Genome Data Sharing (24) advocate for the timely and high-quality sharing of genome data; however, there exists significant variation in data sharing levels among different countries/regions (25). The increased sharing of data may further exacerbate these imbalances and discrepancies. Therefore, a comprehensive understanding of global disparities in shared genomes can enhance objectivity when interpreting genomic data-driven analyses.

During the COVID-19 pandemic from 2020 to 2023, China endeavored to disseminate newly identified SARS-CoV-2 genomes through public databases, encompassing those obtained from the initial COVID-19 patient and the first SARS-CoV-2 genome isolated from the external packaging of cold-chain products (26). However, there is a limited scope for comprehensive assessment and comparison China with other global regions during this timeframe. Therefore, this study aims to comprehensively analyze the global landscape of SARS-CoV-2 genome sharing between 2020 and 2023, focusing on quantity, timeliness, and quality of shared genomes. Additionally, it will specifically examine China’s characteristics in terms of sharing SARS-CoV-2 genomes. Considering the challenges associated with integrating SARS-CoV-2 genome data from diverse repositories, including sequence discrepancies and inconsistent metadata, we opted to utilize the GISAID database as our source for this study due to its extensive collection of SARS-CoV-2 sequences and comprehensive meta-information that surpasses other available resources (9).

## 2 Materials and methods

### 2.1 Data source

The genome and metadata of SARS-CoV-2 were obtained from the GISAID database on October 7, 2024. The total population data of China in 2023 was extracted from National Bureau of Statistics of China.^1^ The standard map [No. GS (2023) 2767] was downloaded without modification from the standard map service website of the National Administration of Surveying, Mapping and Geographic Information. The global and Chinese count of reported cases was sourced from John Hopkins University and the WHO^2^ via the Global Epidemic Analysis and Risk Assessment Platform of China CDC.

### 2.2 Inclusion criteria and data management

The period for genome submission ranged from January 1, 2020 to December 31, 2023. Genomes meeting the criteria of providing complete country of origin information and sampling date. The sampling date no later than the submit date were included in the analysis. The genomes were classified according to the continent and country/region, based on the information provided in the “Location” field of the metadata associated with each genome, indicating the geographical locations where samples were collected. The high-quality whole genome sequences were filtered with a length above 29,000 nt and Ns ≤5% in the entire genome. Genomes from China does not include the Hong Kong Special Administrative Region (SAR), Macau SAR, and Taiwan, China. To investigate the characteristics of shared SARS-CoV-2 genomes in China during 2023, we extracted genomes from GISAID submissions originating from China, covering the period from January 1 to December 31, 2023. The analysis included only individuals classified as “male” or “female” for gender, and age was limited to numeric values ranging from 0 to 200, excluding any symbols other than the decimal point. Genomes meeting both the sex and age criteria were selected for inclusion in the analysis. The prevalence of variants in each year were analyzed based on the information provided in the “Variant” field of the metadata associated with each genome. The proportion of each variant to the total number of shared genomes in each year was calculated.

### 2.3 Statistical analysis

Descriptive analysis was conducted to present the general characteristics of the genomes sharing. Continuous variables were reported using the median and interquartile range (IQR), while categorical variables were presented as counts and proportions. Structured Query Language and Python were used for data cleaning, processing, and generating descriptive statistics, while both Python and GraphPad Prism 9 (GraphPad Software, Inc., LaJolla, CA, United States) were employed for data visualization. Detailed information and codes can be found.^3^

## 3 Results

### 3.1 The sharing of SARS-CoV-2 genomes exhibited worldwide variation in quantity and timeliness across continents

From 2020 to 2023, by searching in the GISAID database, a total of 222 countries/regions actively contributed 16,001,611 SARS-CoV-2 genomes. The annual counts were as follows: 142, 205, 210, and 180 countries/regions with genome contributions amounting to 307,565; 6,205,472; 7,623,101; and 1,865,473, respectively. Compared to the number of reported cases each year, we observed a similar trend between the number of cases and the sharing of genomes. Since the emergence of variants of concern (VOCs), variants of interest (VOIs) and variants under monitoring (VUMs) of the WHO, there has been a global increase in both reported cases and shared genomes. The peak in both case numbers and genome sharing occurred with the Omicron variant in 2022 (Supplementary Figure S1A).

**Figure 1:**
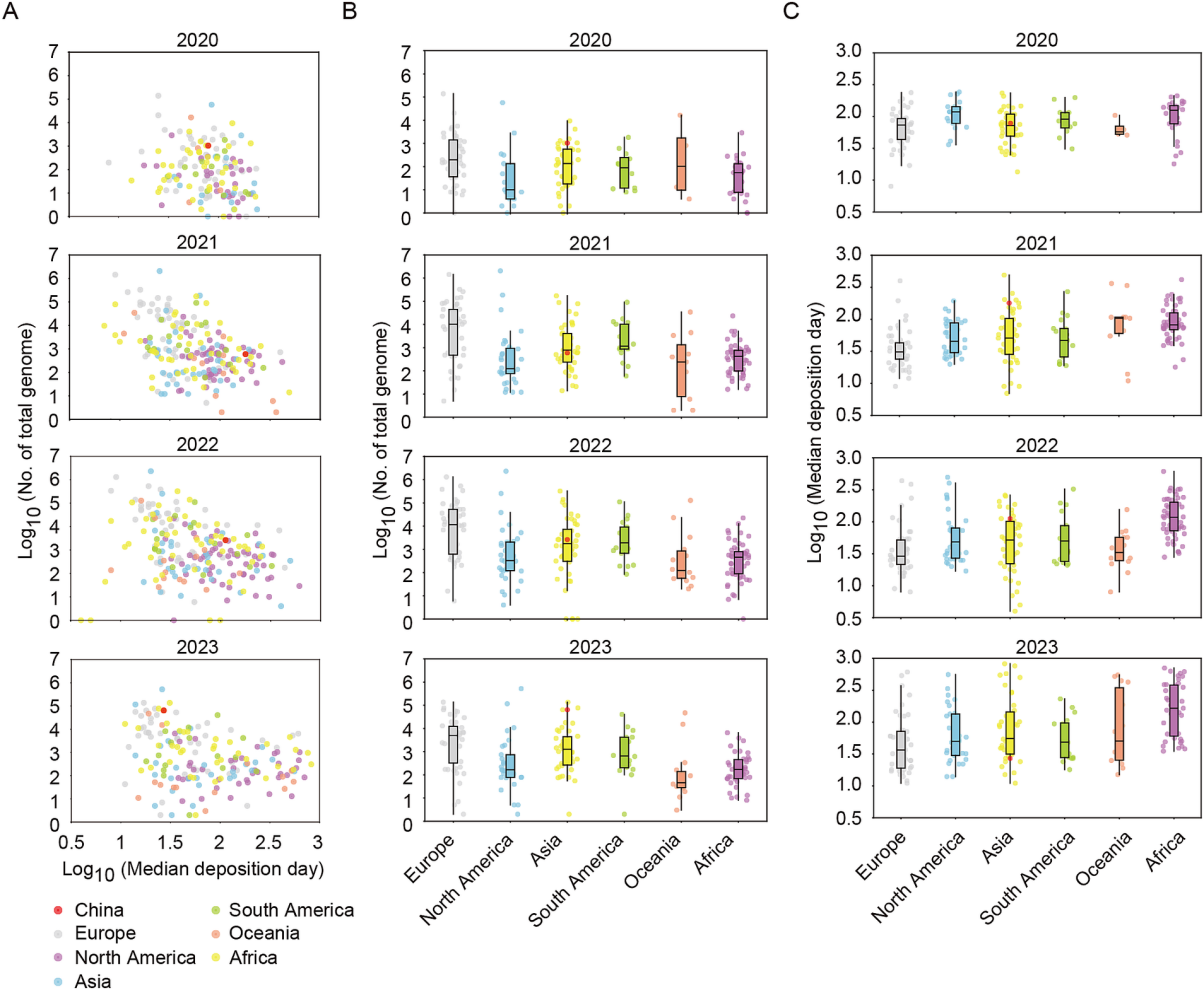
The total number of shared genomes and the median time of genome deposition for each country/region in the period from 2020 to 2023. (A) Scatter plots of total number of shared genomes and the median time of genome deposition for each country or region. (B) Box plots of total number of shared genomes for each country/region divided by six continents. (C) Box plots of median time of genome deposition for each country/region divided by six continents. China was marked in red plot. Median time of genome deposition means the time interval between sample collection and genome sharing.

Next, the numbers and median deposition days of genomes were analyzed across six continents. Certain European, North American, and Asian countries/regions, such as United Kingdom, United States of America, and Singapore et al., exhibited both substantial numbers of shared genomes and short median deposition days (Figure 1A). Overall, the European region consistently contributed a high median number of shared genomes and short median deposition days throughout the years from 2020 to 2023, indicating the continuity and timeliness of genome sharing in Europe (Figures 1B,C). Compared to the prior to 2023, the number and timeliness of shared genomes in China in 2023 (total number: 64302; median deposition days: 27, IQR: 16–52) were far higher than those of countries/regions in Asian (median total numbers: 1258.5, IQR: 265.3–4391.5; median deposition days: 55.3, IQR: 31.5–145.3) and were among the forefront of the world (Figures 1A–C).

### 3.2 The levels in SARS-CoV-2 genome sharing from China remarkable strengthened in 2023

**Figure 2:**
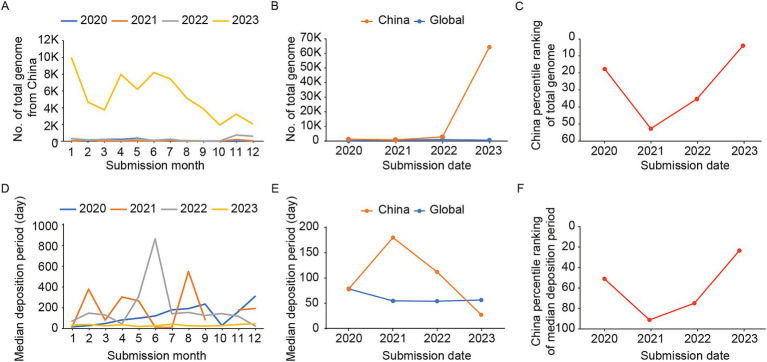
The number of shared genomes and the median time of genome deposition in China from 2020 to 2023. (A) The number of shared genomes in China each month during 2020–2023. (B) The median number of shared genomes in China and among countries/regions worldwide from 2020 to 2023. (C) The relative ranking of China in the world on the number of shared genomes during 2020–2023. (D) The median time of genome deposition in China during 2020–2023. (E) The median time of genome deposition in China and among countries/regions worldwide from 2020 to 2023. (F) The relative ranking of China in the world on the median time of genome deposition during 2020–2023.

To further investigate the features of Chinese SARS-CoV-2 genome sharing, we analyzed the SARS-CoV-2 genomes shared by China on GISAID database. The results showed that the number of shared sequences increased significantly in 2023 compared to the period of 2020–2022 (Figures 2A,B). The highest number of shared sequences occurred in January 2023 (total numbers: 9862), with two small peaks observed in April (total numbers: 7963) and June (total numbers: 8136), followed by a smaller peak in November (total numbers: 3198). Although the number of shared genomes in China from 2020 to 2022 was very similar, the relative ranking of China’s shared genomes in the global countries/regions was lower than the median level in 2021 and 2022, indicating a relatively lower level of genome sharing compared to other regions worldwide. The number of genomes shared by China in 2023 significantly exceeded the median number of shared genomes of countries/regions globally (median total numbers: 379.5, IQR: 94.8–4049.3), and the relative ranking of China had risen to the top 3.89% globally (Figure 2C). Similarly to the global, there were much closer trend between the number of reported cases and the sharing of genomes in China (Supplementary Figure S1B).

The timeliness trend of genome sharing also demonstrates similar patterns, with a more consistent and shorter time interval in 2023 compared to the period of 2020–2022 in China (Figure 2D). In 2023, the median deposition period for genome sharing in China was notably shorter compared to the global median (median deposition days: 56.3, IQR: 28.8–173.0; Figure 2E), ranking among the top 22.78% worldwide (Figure 2F). Therefore, in contrast to the increased global median deposition days for genomes in 2023, China has achieved advancements in both quantity and timeliness.

### 3.3 Noticeable disparity existed in the quality of globally shared genomes

**Figure 3:**
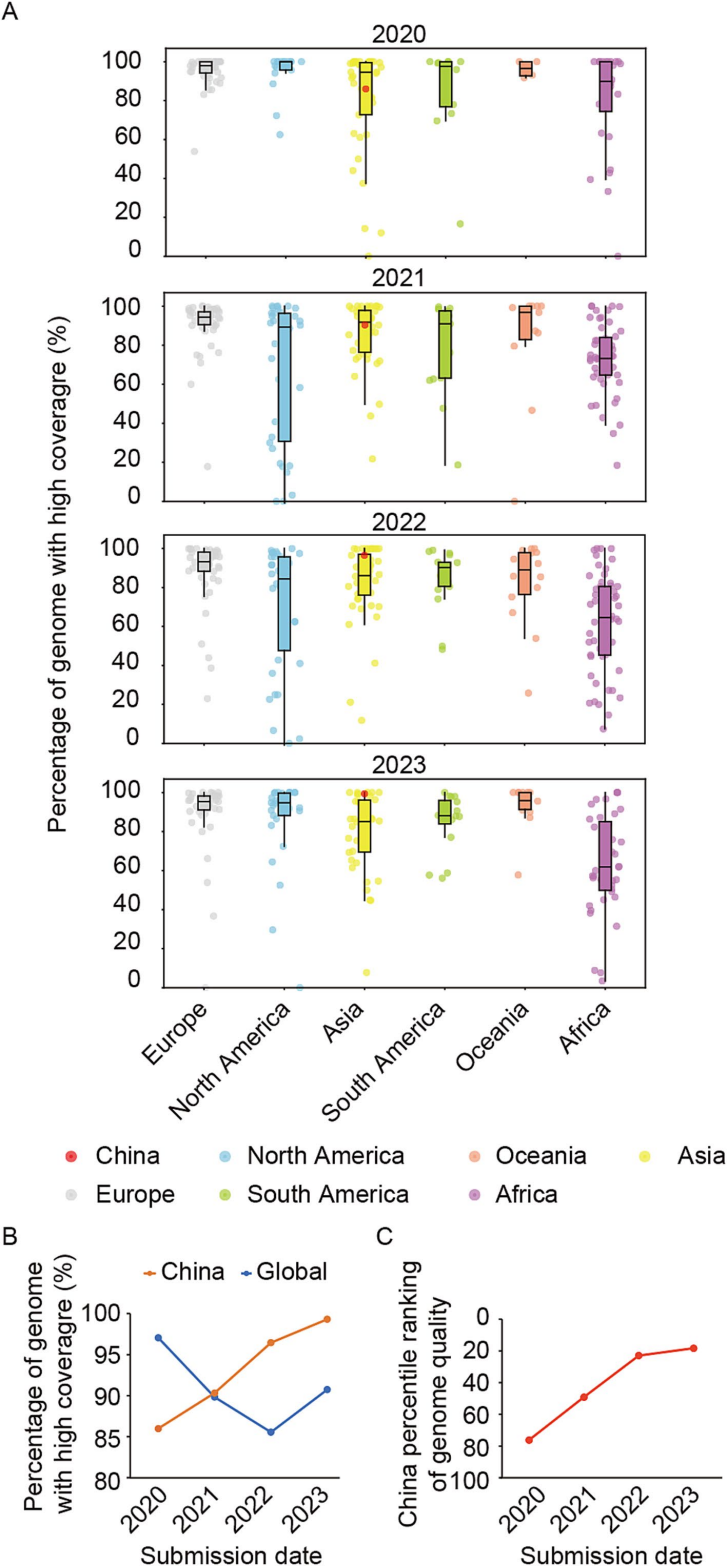
The quality of shared genomes for each country/region in the period from 2020 to 2023. (A) Box plots depict the percentage of genome with high coverage, excluding genomes below 29,000 nt and those with over 5% Ns, for each country or region across six continents. China was marked in red plots. (B) The percentage of genomes with high coverage of China in each year during 2020–2023. (C) The relative ranking of China in the world on the percentage of genomes with high coverage during 2020–2023.

Subsequently, we conducted a comprehensive analysis on the quality of the SARS-CoV-2 genomes shared via GISAID. Genomes with a length above 29,000 nt were selected, while low coverage sequences (Ns >5%) were excluded to obtain high-quality whole genome sequences. The ratio of these high-quality sequences to the total number of sequences was then calculated. The findings indicated that there was high median ratio and minimal degree of dispersion in genome quality among the shared genomes from European countries/regions from 2020 to 2023, demonstrating consistently high quality in European countries/regions (Figure 3A). Conversely, African exhibited a lower median ratio and a wider dispersion, suggesting an overall lower quality of genomic sequences with significant disparities among countries/regions. Notably, the quality of genomes shared from China in 2020 falls below the median level for Asia and globally. However, it demonstrated steady improvement over subsequent years, surpassing the median levels of both Asia and global by 2022 (Figures 3A,B). By 2023, the relative ranking of quality for shared genomes by China has risen to the top 17.78% worldwide (Figure 3C).

### 3.4 The genomic metadata shared in China in 2023 exhibited distinct characteristics

**Figure 4:**
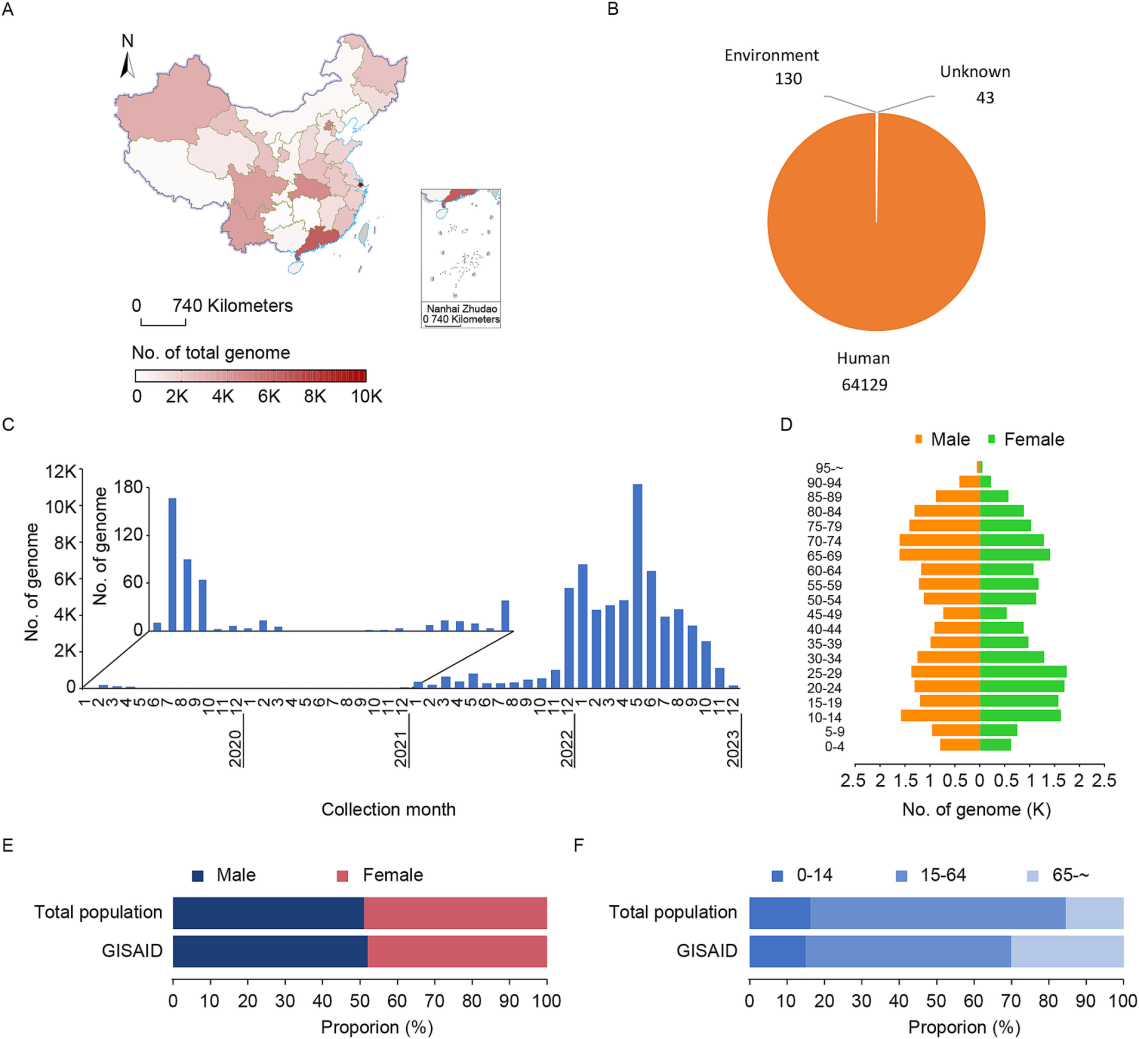
Characteristics of shared SARS-CoV-2 genomes in China in 2023. (A) The geographical distribution of SARS-CoV-2 genomes shared from China in 2023. The 395 genomes that lacked province information were excluded. (B) The host distribution of SARS-CoV-2 genomes shared from China in 2023. (C) The temporal distribution of collection dates for genomes sampled from the Chinese population and shared in 2023. (D) The distribution of sex and age of genomes sampled from the Chinese population in 2023. The 10,767 genomes lacking standard sex and age information were excluded from the analysis. (E) The sex distribution Chinese total population in 2023 between the total population and GISAID. (F) The age distribution comparison of the Chinese total population in 2023 between the total population and GISAID.

In 2023, China made great efforts in genome sharing. Although all provinces in China shared genomic sequences, there were regional differences. Shanghai Municipality, Guangdong Province, and Beijing Municipality shared the most sequences, with 9,970, 6,310, and 4,693, respectively, (Figure 4A). From the species composition of the shared sequence, the vast majority were human samples (total number: 64,302), followed by environmental samples (total number: 130), and 43 samples of unidentified species (Figure 4B), suggesting that China’s monitoring strategy in 2023 focused primarily on population surveillance with secondary emphasis on environmental monitoring. The sampling collection dates of population data showed a concentration after December 2022 with three prominent peaks: December 2022 to January 2023, May to June 2023, and August to September 2023 (Figure 4C), indicating that concentrated data sharing during these periods may be associated with clustered outbreaks. Gender distribution was evenly balanced, with 52.06% male and 47.94% female. The gender composition spanned all age groups but was mainly concentrated in the age ranges of 10–29 and 65–74 (Figure 4D). A comparison between GISAID’s sampled population distribution and China’s total population revealed similar gender ratios but marked differences in age structure (Figure 4E, male: total population vs. GISAID: 51.10% vs. 52.06%). Notably, a significantly higher proportion of individuals over 65 years old among GISAID’s shared genomic data (Figure 4F, total population vs. GISAID: 15.40% vs. 30.01%), potentially attributed to specific surveillance targeting this older adult population.

## 4 Discussion

This study systematically analyzed and compared the differences in SARS-CoV-2 genome sharing among countries/regions across continents during the pandemic from 2020 to 2023. The study findings demonstrated that the European countries/regions had superior performance in terms of the quantity, timeliness, and quality of shared genomes compared to other countries/regions. Starting from 2023, China has made significant improvements in all these aspects, with full coverage of provinces, timely sharing, and widespread monitoring of the population. The findings of our research suggested an expansion of data sharing capacity during the SARS-CoV-2 pandemic. It will be critical in containing and responding to future pandemics caused by novel pathogens.

Our analysis reveals substantial disparities in global genome sharing among different countries/regions. In view of the extensive incorporation of genomic data within GISAID, conducting comprehensive genome quality control measures, such as identifying frameshifts, posed a challenge. In our study, a threshold exceeding 29,000 nt coverage along with less than 5% Ns were utilized to define high quality whole genome sequencing. Notably, we found that Europe demonstrated a higher level of genome sharing compared to other continents, while Africa exhibits relatively lower performance. These variations may stem from diverse factors encompassing discrepancies in sequencing capacities as well as policy and financial support across nations. Consistent with our findings, most East African Community nations encountered challenges including insufficient local NGS equipment, limited bioinformatics expertise, inadequate computational resources, and ineffective data-sharing mechanisms (27). However, the Public Health England has been recognized as an early leader at a national level for employing high-throughput sequencing for pathogeny surveillance (28, 29). The quality of related metadata, in addition to sequence quality, was also deemed important. A previous study revealed a prevalent occurrence of incomplete metadata worldwide for GISAID sequences. Specifically, approximately 63% of the sequences lacked demographic information, 84% were devoid of sampling strategy details, and patient-level clinical information was missing in over 95% of the cases (30). One limitation of our study is that we used the sample collection location as the country for analyzing genome sharing levels. However, there may be potential bias in assessing actual sharing performance among countries/regions due to inter-regional scientific projects leading to differences between the submitting country and sample collection country/region. Regardless, the COVID-19 pandemic undeniably propels pathogen whole-genome sequencing endeavors and facilitates data sharing.

The growing prevalence of shared genomes presents several challenges, such as the management of extensive public databases, and the issue of duplicate data uploading. The RCoV19 database, for instance, possesses the capability to integrate and eliminate redundant genomes as well as annotate database sources (31, 32). RCoV19 offers a comprehensive integration of data and identifies the same genome sequences submitted to different sources by comparing key meta information (virus name, collection date, and location) as well as sequences after removing Ns and unifying the letter case (9). Besides RCoV19, the VirusDIP (33), ViruSurf (34), and CoV-Seq (35) databases also perform data integration and de-redundancy processing. However, it is worth noting that ViruSurf and CoV-Seq have not been updated since January 2022 and September 2020, respectively. On the other hand, VirusDIP integrates data from GISAID, GenBank, and CNGBdb but does not include information from GenBase and NCNSTRSS. There databases’s efforts have greatly improved the accessibility of comprehensive datasets for users. Even then, data incompleteness is an unavoidable limitation for integration, potentially resulting in information loss due to format discrepancies across different databases. Moreover, this limitation also hampers genomic surveillance as the representation of virus distribution may be skewed due to information incompleteness on local or travel-related cases in the majority of genomes.

Our findings demonstrate the substantial progress made by China in sharing SARS-CoV-2 genomic data. As demonstrated in a previous study, achieving a sequencing turnaround time of less than 21 days could serve as a benchmark for effective SARS-CoV-2 genomic surveillance (36). Here, the median turnaround time for China in 2023 was 27 days, which closely approached the aforementioned threshold, indicating a significant improvement and underscoring the imperative for sustained efforts. Before 2023, China’s robust prevention and control measures, coupled with successful vaccination campaigns, led to a minimal incidence of cases, with the majority of domestic outbreaks attributed to imported infections from overseas. This correlation was consistent with the limited number of shared genomes by China during the period spanning 2020–2022. Due to the adjustment of COVID-19 prevention and control policy at the end of 2022 (37), coupled with the continuous evolution of Omicron variant, there was an increase in reported cases can be observed in China. Simultaneously, there has been a corresponding rise in shared genomes showing consistency. Upon analyzing the temporal distribution of shared genomes of China sampled in 2023, we observed three distinct peaks in the epidemic: December 2022 to January 2023, May 2023 to June 2023, and August 2023 to September 2023. Remarkably, these peaks closely align with the positive rate of COVID-19 among influenza-like cases reported by the China CDC (38). However, there were variations in peak intensity, particularly observed during the May to June 2023 peak. Therefore, genomic data sharing can only serve as a reference for rough estimating the actual epidemic. Given that China made adjustments to its epidemic prevention and control policy at the end of 2022, timely sharing of genomic data will facilitate comprehensive and expeditious analysis of circulating variants within China by both domestic and international researchers.

All in all, the analysis of SARS-CoV-2 genomic data sharing during 2020–2023 reveals significant advancements, particularly in countries like China. The efforts made by China and the global community in sequencing and sharing genome sequences during the COVID-19 pandemic undeniably contribute to advancing the One Health objective’s requirements of ensuring discoverable, accessible, interoperable, and reusable data (39). These endeavors also facilitated further researches and the evidence-based policies in response to the spread of VOCs and VOIs (40–44). However, it is crucial to acknowledge the variations in the extent of global genomic data sharing across different regions worldwide. The timely sharing of data is crucial for effectively addressing the current COVID-19 situation, as well as enhancing our preparedness for future outbreaks of emerging pathogens. Further efforts are warranted to address the disparity in global genomic data sharing and establish a universally standardized platform for data utilization, in order to promote scientific collaboration and advance research progress.

## Data Availability

Publicly available datasets were analyzed in this study. This data can be found at: https://github.com/SongqiChen/covid19-genome-sharing-analysis.

## Footnotes

^1^ https://data.stats.gov.cn/english/easyquery.htm?cn=C01

^2^ https://covid19.who.int/data

^3^ https://github.com/SongqiChen/covid19-genome-sharing-analysis
