# Supplementary material for: Trends and impacts of SARS-CoV-2 genome sharing: a comparative analysis of China and the global community, 2020–2023
Source: Front Public Health. 2024 Nov 20;12:1491623. doi: 10.3389/fpubh.2024.1491623 (PMC11614776; doi:10.3389/fpubh.2024.1491623)
Supplement: Supplementary file 1 [file Supplementary_file_1.docx]

Supplementary Material

## Supplementary Figure


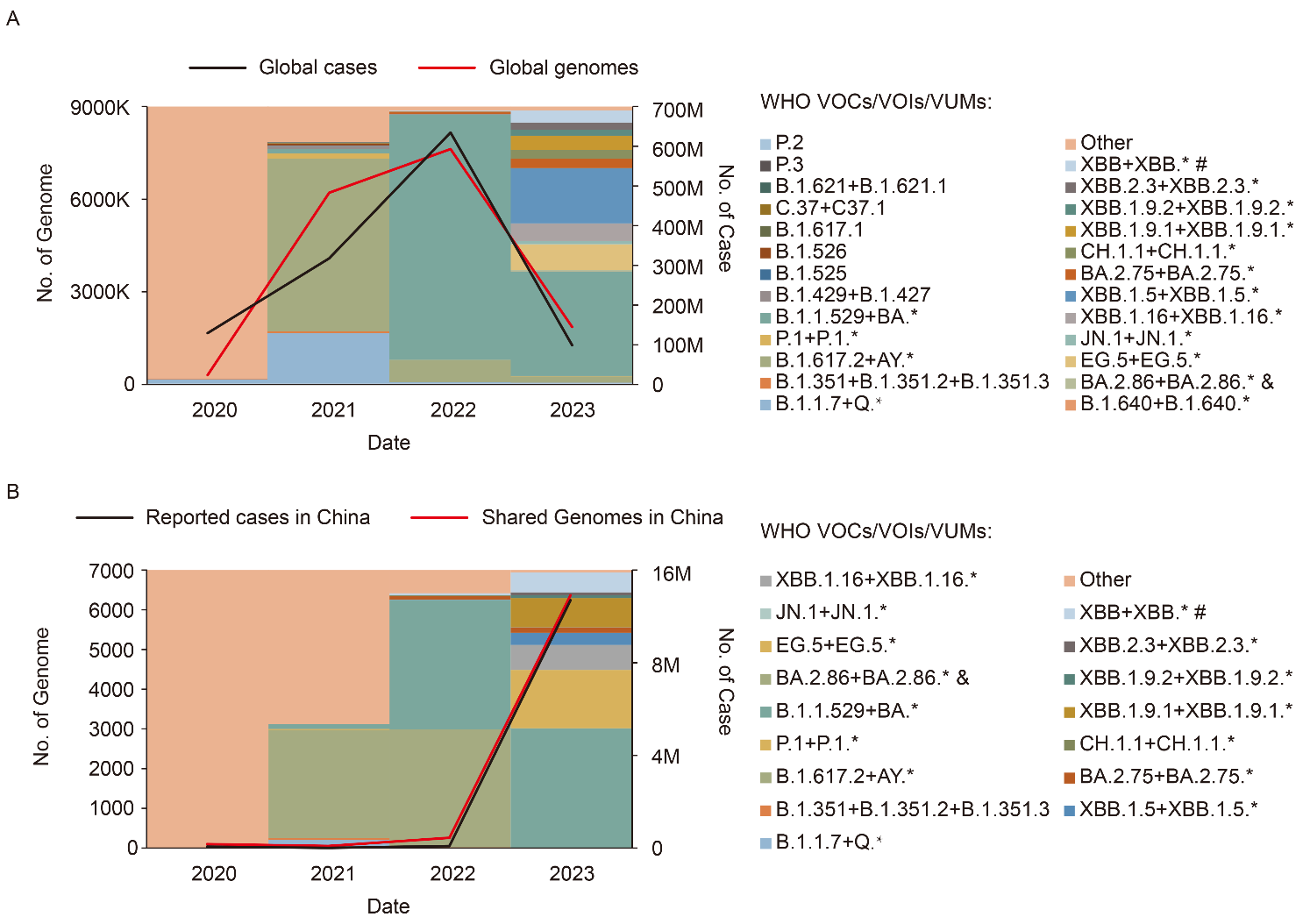


**Supplementary Figure 1.** **The epidemiological and variant prevalence landscape from 2020 to 2023.** The number of shared genomes, reported cases and variants prevalence **(A)** globally and **(B)** in China during 2020-2023. *, a wildcard for conducting searches; #, excluding XBB.1.5, XBB.1.16, XBB.1.9.1, XBB.1.9.2, XBB.2.3; &, excluding JN.1, JN.1.*.
